# Limited Cutaneous Leishmaniasis as Ulcerated Verrucous Plaque on Leg, Tucson, Arizona, USA^1^

**DOI:** 10.3201/eid2906.230125

**Published:** 2023-06

**Authors:** Caitlyn B. Dagenet, Mitchell S. Davis, Sean Murphy, Rebecca Thiede, Keliegh S. Culpepper, Mohammad Fazel

**Affiliations:** University of Arizona College of Medicine, Tucson, Arizona, USA (C.B. Dagenet, R. Thiede, M. Fazel);; University of California, San Francisco, California, USA (M.S. Davis);; Tucson ER and Hospital, Tucson (S. Murphy); DermPath Diagnostics, Tucson (K.S. Culpepper)

**Keywords:** cutaneous leishmaniasis, leishmaniasis, leishmania, parasites, sand flies, dermatology, case report, ulcerated verrucous plaque, leg, zoonoses, Tucson, Arizona, United States, *Suggested citation for this article*: Dagenet CB, Davis MS, Murphy S, Thiede R, Culpepper KS, Fazel M. Limited cutaneous leishmaniasis as ulcerated verrucous plaque on leg, Tucson, Arizona, USA. Emerg Infect Dis. 2023 Jun [*date cited*]. https://doi.org/10.3201/eid2906.230125

## Abstract

We report a 34-year-old man who had a nonhealing, verrucous plaque with central ulceration on the lower leg. This case-patient is a rare example of endemic limited cutaneous leishmaniasis in Tucson, Arizona, USA. Clinicians should be aware of this disease because its manifestations can vary for individual patients.

Cutaneous leishmaniasis (CL) is the most common form of leishmaniasis and typically manifests as solitary painless lesions at the site of sand fly bites. This disease is generally acquired in specific habitat environments conducive to its sand fly vector and reservoir hosts (*1*). Most cases of CL diagnosed in the United States are attributed to persons who travel (e.g., military, international workers) to disease-endemic countries (e.g., India, Nepal, Brazil, Sudan, and Bangladesh) (*1*,*2*)

Although the United States was classified as endemic for leishmaniasis by the World Health Organization in 2020, the state of Arizona has had few documented cases (*1**‒**4*). A Medline search of authors showed only 3 cases of CL have been reported in Arizona (*1*,*2*,*4*). Of those case-patients, only 1 truly autochthonous case-patient did not report recent travel to a disease-endemic location (*1*,*2*,*4*). Despite the rarity of human CL cases, sand fly vectors (e.g., *Lutzomyia anthophora*) and hosts (e.g., *Neotoma albigula* [white-throated woodrat]) can transmit New World *Leishmania* spp. and have been documented in Arizona (*5*,*6*). We report a 34-year-old man who had a nonhealing, verrucous plaque with central ulceration on the lower leg.

Consent for publication of all patient photographs and medical information was provided by the authors at the time of article submission. The patient gave consent for their photographs and medical information to be published in print and online and with the understanding that this information mighty be publicly available.

The patient had no major medical history and no history of skin cancer or immunosuppression. He came to the dermatology clinic in Tucson, Arizona, because of a new onset of a painless lesion on the right lateral lower leg. The lesion was present for 6 weeks before the visit. Physical examination demonstrated a 2.1 × 1.1 cm solitary, nonhealing, verrucous plaque with central ulceration on the right proximal lateral pretibial region (Figure). The patient reported considerable time outdoors and recreational activities, such as mountain biking and hiking. Those activities occurred in Arizona; he specifically denied a history of recent travel outside of the United States. He also denied any trauma to the site of infection.

**Figure:**
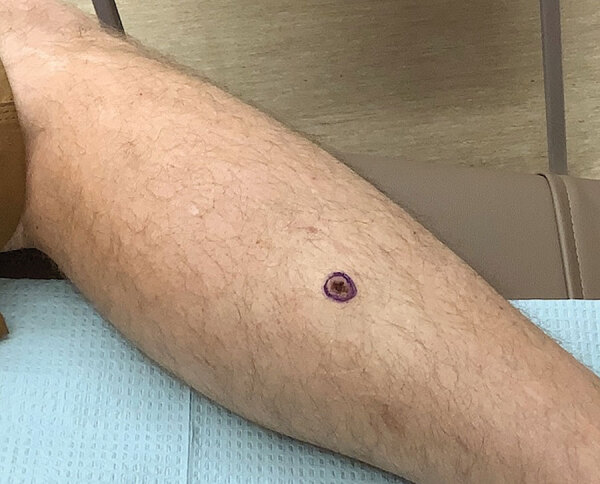
Limited cutaneous leishmaniasis as ulcerated verrucous plaque on leg of patient in Tucson, Arizona, USA. A solitary, nonhealing plaque with central ulceration is shown on the right proximal lateral pretibial region.

Shave biopsy and subsequent staining with hematoxylin and eosin showed focal ulceration with mixed acute, chronic, and granulomatous inflammatory infiltrate and minute organisms in the cytoplasm of histocytes favoring parasitized histiocytic infection. The organisms were negative by staining with periodic acid–Schiff. Microscopic examination was then completed by the Centers for Disease Control and Prevention, and small intracellular organisms compatible with protozoal amastigotes were observed within macrophages. Immunohistochemical analysis subsequently confirmed a diagnosis of leishmaniasis. The patient was referred to an infectious disease department, where observation was recommended because he had a solitary cutaneous lesion that was removed in entirety by biopsy. He also had no mucosal involvement.

In this case, the new-onset ulcerated plaque was initially most concerning for infectious versus neoplastic etiology. Although an infectious process was considered, CL was not on the original differential diagnosis given the rarity of this diagnosis in Arizona. This case helps demonstrate that leishmaniasis must be a consideration when patients in Arizona have verrucous papules or plaques.

Most autochthonous cases of CL in the Unites States have been in Texas (*6*). In 2018, a study in Texas identified 69 novel cases of leishmaniasis and 22 having documented speciation (all *L. mexicanca*) (*4*). CL is probably underreported because reporting is not required in most states, and a high rate of misdiagnosis can be presumed (*4*,*6*). Sand flies capable of transmitting *L. mexicana* have been reported in several states (*7*).

CL treatment decreases the risk for mucosal dissemination, accelerates healing of the lesion, decreases the risk for relapse, and decreases illness. Relapse of cutaneous disease (leishmaniasis recidivans) can occur several years after resolution of the primary lesion in treated and untreated patients (*9*). Treatment options for CL include systemic miltefosine (for *Leishmania* [*Viannia*] spp.), ketoconazole (for *L. mexicana*), and fluconazole or local therapy (antimonial drugs, topical paromomycin, or liquid nitrogen therapy) (*9*).

Treatment of leishmaniasis is determined by species, risk for mucosal dissemination, size, number, and location of lesion(s). Limited CL does not require further treatment if the findings self-resolve. CL can spontaneously resolve within 2 months to a year. CL that has >5 lesions; an area >5 cm; is on the face, toes, fingers, or genitalia; or has a duration >6 months might require treatment to prevent relapse or progression to mucosal disease (*2*). Other complications of CL include secondary infections and scarring (*8*).

CL treatment decreases the risk for mucosal dissemination, accelerates healing of the lesion, decreases the risk for relapse, and decreases illness. Relapse of cutaneous disease (leishmaniasis recidivans) can occur several years after resolution of the primary lesion in treated and untreated patients (*9*). Treatment options for CL include systemic miltefosine (for *Leishmania* [*Viannia*] spp.), ketoconazole (for *L. mexicana*), and fluconazole or local therapy (antimonial drugs, topical paromomycin, or liquid nitrogen therapy) (*9*).

In conclusion, CL is an increasing concern in the United States where endemic cases have been identified, most prominently the southern and southwestern regions. Our case adds to the short but increasing list of documented CL cases in Arizona. Placing CL in the differential diagnosis for new-onset verrucous plaques of unknown etiology in local disease-endemic areas of the United States is prudent. Limited CL can be managed conservatively with monitoring for recurrence once the solitary lesion has been removed, but further treatment might be necessary depending on the manifestations in an individual patient.
